# Using Network Science to Examine Temporal Relationships between 24-Hour Movement Behaviors and Depression During the Transition to College: Protocol for a Prospective Cohort Study

**DOI:** 10.21203/rs.3.rs-9239716/v1

**Published:** 2026-04-19

**Authors:** Denver M. Y. Brown, Carah D. Holesovsky, Michael S. Vitevitch, Kelsie T. Forbush

**Affiliations:** Kansas State University; Kansas State University; University of Kansas; University of Kansas

**Keywords:** sleep, sedentary behavior, physical activity, Fitbit, compositional data analysis, emerging adulthood, intensive longitudinal methods, precision psychiatry, multilevel vector autoregression

## Abstract

**Background:**

The transition to college coincides with the peak age of onset for depression and is marked by substantial changes in 24-hour movement behaviors (sleep, sedentary behavior, physical activity). Although these behaviors are modifiable and increasingly implicated in depression risk, most studies rely on cross-sectional, self-reported data and aggregate symptom scores, limiting insight into temporal dynamics and symptom-level heterogeneity. Integrating compositional modeling of 24-hour time use data with network approaches to psychopathology may clarify when and how specific behavioral patterns relate to distinct depressive symptoms during this high-risk developmental window.

**Methods:**

The College Adjustment, Lifestyle and Mental Health (CALM) Study is a 16-week prospective cohort study of 144 first-year undergraduate students (Mage = 18.2 ± 0.4 years; 54.9% female) during their first academic semester. A hybrid panel-burst design combined five monthly panel surveys assessing psychosocial and behavioral factors with five 7-day intensive daily diary assessment bursts distributed across a 108-day period. During each burst, participants completed daily diary adaptations of the PHQ-8 and GAD-7 to capture depressive and anxiety symptoms, along with other contextual variables. Sleep, sedentary behavior, light physical activity, and moderate-to-vigorous physical activity were assessed daily using Fitbit Charge 6 devices. Primary analyses will model daily 24-hour movement behaviors using compositional data analysis to account for the constrained nature of time-use data and integrate these compositions into multilevel vector autoregressive models to estimate within-person temporal, contemporaneous, and between-person associations with individual depressive and anxiety symptoms. Secondary analyses will examine symptom-behavior network differences across movement behavior profiles and assess stability of symptom-behavior networks across the first college term to identify periods of heightened risk for symptom onset and progression.

**Discussion:**

By embedding wearable-derived 24-hour movement behavior compositions within dynamic symptom networks, this study advances precision behavioral psychiatry beyond aggregate depression scores by identifying which behaviors are most strongly linked to specific symptoms, for whom these associations differ, and when risk intensifies during the transition to college. The hybrid panel-burst design further provides a foundation for future predictive modeling of individual risk trajectories and the development of just-in-time adaptive interventions leveraging passive sensing to support early detection and prevention of depression in emerging adults.

## Introduction

College student mental health represents a substantial and growing public health concern. Estimates suggest that between 12% and 50% of college students worldwide experience a mental disorder,^1,2^ a prevalence that exceeds their non-college-attending peers.^2^ Mental health problems are particularly pronounced during the first two years of college, especially during the freshman year, as students navigate a major developmental and contextual transition.^3–5^ This period coincides with the peak age of onset for depression (~ 19.5 years),^6^ further elevating vulnerability among college students. Although symptoms often attenuate by junior year, recent U.S. data indicate that clinically significant depressive symptoms have persisted at rates exceeding 36% since 2018.^7^ Given that nearly two-thirds of U.S. high school graduates enroll in college,^8^ depression during this life stage has important population-level implications, particularly as early-onset depression is associated with elevated risk for recurrence and long-term functional impairment in adulthood.^9^

A substantial body of research has identified modifiable lifestyle behaviors as relevant determinants of mental health during this developmental period.^10–17^ As a result, behavioral approaches are increasingly considered as scalable complements to, or alternatives for, traditional psychotherapy and pharmacological treatments.^18–21^ Physical activity (PA), in particular, has received considerable attention due to its associations with reduced depressive symptoms in young people, including during the transition to college,^13^ with some evidence supporting a causal effect.^22^ However, an exclusive focus on PA provides an incomplete account of daily behaviors that occur within a 24-hour window, as other time-use domains such as sleep and sedentary behavior (SB) also play important roles in shaping mental health.

Over the past decade, there has been a shift from examining PA, SB, and sleep – collectively referred to as 24-hour movement behaviors – in isolation toward an integrated approach that examines all of these behaviors together.^23,24^ The 24-hour movement paradigm emphasizes that the “whole-day” matters for health and acknowledges these behaviors as interdependent components of a fixed 24-hour period. This perspective is reflected in the Canadian 24-Hour Movement Guidelines for Adults, which were the first to provide evidence-based recommendations for how much moderate-to-vigorous PA (MVPA), sleep, and SB adults should engage in over the course of a whole day.^25^ Studies have shown that meeting a greater number of these recommendations is associated with lower levels of depressive symptoms and depression risk.^10,26^ However, reliance on threshold-based guideline adherence obscures substantial within- and between-person variability in movement behavior patterns. For example, individuals who accumulate 150 minutes of MVPA per week are classified as having met this guideline, whereas those accumulating 149 minutes are grouped with individuals reporting no MVPA at all, despite likely meaningful differences in exposure and associated mental health risk based on established dose-response associations.^27^ These limitations are further compounded by widespread use of self-reported measures and cross-sectional designs,^10^ which restrict inferences about temporal dynamics linking 24-hour movement behaviors and depression.

Accurately modeling 24-hour time-use data, therefore, requires analytic approaches that explicitly account for both the continuous nature and interdependence of daily movement behaviors.^28^ Compositional data analysis (CoDA) is an innovative approach commonly used in the field of time-use epidemiology that accounts for the interdependence of time-use components (i.e., PA, SB, sleep) within a constrained 24-hour window, in which time allocated to one behavior reduces time available for others (e.g., increasing PA inherently takes away from time spent in SB and sleep).^29,30^ Unlike traditional statistical models, CoDA accommodates this perfect collinearity in time-use data, minimizing bias and enhancing accuracy.^28,29^ Device-based measures such as actigraphy are particularly well suited to CoDA, as they provide continuous estimates of time spent engaging in sleep, SB, and PA across the entire day. Despite the introduction of CoDA to movement behavior research over a decade ago,^31^ most studies continue to rely on short observational windows (e.g., 7-day accelerometer wear period), obscuring our understanding of the dynamic and evolving nature of these behaviors in relation to depression.^10^

Greater temporal resolution may be critical for elucidating pathways linking 24-hour movement behaviors and depression. Although device-based assessments address key limitations of self-reported PA measures,^32,33^ much of this work has relied on aggregated, low-resolution summaries (e.g., weekly averages) assessed at widely spaced intervals (e.g., baseline, 3 months, 6 months). Only recently have studies begun to leverage high-resolution time series data (i.e., daily summaries across several months) from consumer wearables (e.g., Fitbit, Apple Watch) to characterize long-term behavioral patterns.^34,35^ Employing CoDA with intensive longitudinal device-based data and frequent assessments of depressive symptoms (e.g., multiple weeks of daily diaries) could provide novel insights into within-person associations between 24-hour movement behaviors and depressive symptoms, and how these dynamic relationships evolve as students navigate newfound stressors during the transition to college, while accounting for the inherent measurement properties of 24-hour time-use data.^36^

Advances in analytic methods for time-use data also coincide with broader shifts in how mental disorders are conceptualized. Traditional psychiatric frameworks largely rely on a biomedical approach, viewing disorders as latent disease entities wherein symptoms reflect underlying disease process or common cause.^37^ This approach often models mental disorders as a binary outcome or a sum of symptom severity, treating symptoms as interchangeable and independent.^38,39^ However, symptom presentation varies widely, even within diagnostic categories.^40^ Recognizing this, recent work has reconceptualized mental disorders as emerging from networks of causally interacting symptoms, wherein symptoms influence one another both directly and indirectly, rather than reflecting a common latent cause.^41^ This approach facilitates the study of symptoms as part of a complex and dynamic system involving many interacting elements, offering novel implications for understanding the etiology and course of mental disorders.^42^

Extending mental health symptom networks to incorporate non-symptom risk and protective factors may further enhance their utility. Studies have called for integration of behaviors such as sleep, SB, and PA to better understand the dynamic factors influencing disorder etiology.^43,44^ Doing so may help identify behaviors that exert disproportionate influence on specific symptoms, in addition to revealing self-reinforcing pathways (e.g., insufficient sleep → fatigue → sadness → loss of interest) that represent actionable intervention targets. Although network-based approaches hold promise for advancing precision prevention and treatment strategies, their application to 24-hour movement behavior research for understanding mental health remains limited to date. Addressing this gap will require intensive longitudinal designs that combine extended time-series assessments of 24-hour movement behaviors with repeated measurement of depressive symptoms.

The purpose of this paper is to describe the protocol and baseline characteristics of the College Adjustment, Lifestyle and Mental Health (CALM) Study, an intensive longitudinal cohort study designed to examine dynamic relationships between device-assessed 24-hour movement behaviors and depressive symptoms during the transition to college. Specifically, this study aims to characterize day-to-day associations between 24-hour movement behaviors and individual depressive symptoms, describe movement behavior patterns associated with elevated risk for depressive symptom onset and progression, and identify periods of heightened vulnerability during the transition to college.

## Methods

### Study Design

The CALM Study was a prospective cohort study that followed first-year undergraduate students for 108 days (September 2-December 19, 2025) during their transition into their first academic term at university. Participants were onboarded between August 25 and September 5, 2025. The study employed a hybrid panel-burst design to capture both longer-term changes across the semester and short-term, day-to-day fluctuations in depressive symptoms and 24-hour movement behaviors. This design is consistent with recommendations for measurement-burst designs that integrate intensive short-term assessment within longer-term longitudinal follow-up to distinguish within-person dynamics from broader developmental change.^45,46^

Participants (N = 144) completed five panel surveys administered at four-week intervals following baseline to assess broader psychosocial and contextual factors across the semester. In parallel, five 7-day intensive daily assessment bursts were distributed across the study period (Days 1–21, 22–42, 43–62, 63–82, and 90–108). The intensive burst design was selected as it optimally balances long study windows with reduced participant burden in smartphone-based research.^47^ Burst timing was randomized within predefined calendar windows and did not align systematically with panel survey administration. No bursts were scheduled during Fall break (Days 83–89) to avoid confounding assessments with atypical routines and travel-related disruptions.

**Figure 1:**
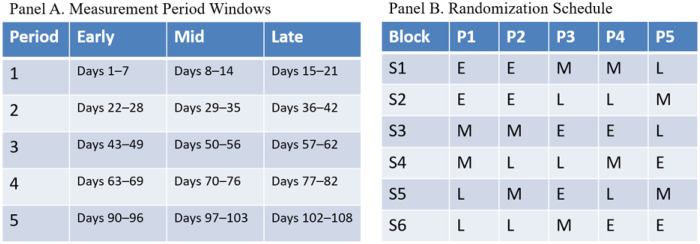
Daily diary burst timing (Panel A) and randomization schedule (Panel B). Panel A displays the five 7-day burst periods and their timing within the 16-week study, with each period comprising early, mid, and late windows. Panel B illustrates the randomized allocation of burst timing (early, mid, late) for each of the six cohorts (S1-S6) across the five data collection periods (P1-P5).

To minimize systematic timing effects across the academic term while preserving calendar balance, burst timing was randomized using a constrained block allocation schedule. The 108-day study was divided into five consecutive calendar periods (four 20–21 day windows during the semester and a final 19-day window following fall break). Each period contained three potential 7-day burst windows corresponding to Early, Mid, and Late segments of the period (Fig. 1). Participants were assigned to one of six pre-specified burst sequences (S1-S6), each representing a unique ordering of Early, Mid, and Late burst timing across the five study periods. A fixed set of sequences was used to ensure exact balance across burst positions while maintaining feasibility within the study platform. This allocation scheme was developed to satisfy the feasibility and balance constraints outlined in Table 1.

### Participants and Recruitment

Participants were first-year undergraduate students enrolled at Kansas State University during the Fall 2025 semester. Eligibility criteria included: (1) age ≥ 18 years at enrollment; (2) enrollment in their first college term immediately following high school graduation; (3) ownership of a compatible smartphone to complete daily surveys and synchronize wearable data; (4) residence in on-campus housing; and (5) absence of mobility impairments that would substantially alter gait patterns, as many research-grade accelerometer- and consumer wearable-based PA algorithms are validated in ambulatory populations with typical gait patterns. Students participating in varsity or club athletics were excluded to reduce heterogeneity in structured PA exposure and related scheduling demands that may differ systematically from those of the general student population.

Recruitment targeted the incoming freshman class (approximately 4,000 students) prior to and during the start of the Fall 2025 semester using a multi-pronged outreach strategy. Specifically, recruitment included: (1) printed handouts containing a QR code linking to an online eligibility screener, distributed in onboarding packets during in-person orientation visits in June 2025; (2) email announcements sent to the incoming class in July 2025; (3) advertisements posted on the Canvas page for incoming School of Health Sciences students during Summer 2025; (4) in-person tabling at a university-wide orientation event during move-in week; (5) in-person announcements and tabling at School of Health Sciences orientation events; and (6) advertisements posted on Psychology 101 course shells. Interested students completed an online screening survey to determine eligibility and were contacted promptly if eligible. Follow-up correspondence occurred in early August to schedule baseline laboratory visits.

### Sample Size Calculation

The present study was powered based on the intensive burst (daily diary) component rather than the monthly panel surveys, as primary hypotheses focused on within-person daily associations between 24-hour movement behaviors and depressive symptoms. Sample size considerations for intensive longitudinal designs are influenced by the number of participants, number of repeated observations per participant, and expected within-person effect sizes.^48,49^ Daily diary studies examining PA and affect, including negative affect items that reflect depressive symptoms, have typically observed small within-person effects.^50–53^ Assuming a small effect size (d = 0.20) and approximately 30–35 daily observations per participant across the five bursts, simulation-based estimates suggested that approximately 107 participants would provide 80% power to detect within-person effects.^54,55^ Allowing for up to 30% attrition across the semester, we aimed to recruit 139 participants.

### Baseline Cohort Characteristics

A total of 144 students were enrolled in the study and completed baseline assessments. Baseline characteristics are presented in Table 2. Participants were approximately 18 years of age and slightly more likely to identify as female (54.9%) than male. The sample was predominantly White (78.5%), with 16.7% identifying as first-generation college students. Students were enrolled across multiple academic colleges within the university, most commonly Health and Human Sciences (29.9%), Engineering (27.8%), and Arts and Sciences (20.8%). At baseline, 80.6% of participants reported no prior mental health diagnosis, 16.0% reported a current diagnosis, and 2.1% reported a previous diagnosis that was not current. All participants provided informed consent prior to participation. The study protocol was approved by the Kansas State University Institutional Review Board (IRB #12780).

### Measures

#### Depressive Symptoms (Primary Outcome).

Depressive symptoms were assessed using both panel and intensive daily diary measures. At baseline and monthly panel assessments (Weeks 0, 4, 8, 12, and 16), symptoms were measured using the 8-item Patient Health Questionnaire (PHQ-8),^56^ a widely validated instrument with strong psychometric properties in college and emerging adult populations.^57,58^ The PHQ-8 excludes the suicidality item from the original PHQ-9,^59^ an approach commonly used in large-scale epidemiologic and community-based research. The suicidality item was excluded to align with the study’s safety monitoring protocol for periodic, self-administered assessments without real-time clinical follow-up. Prior studies indicate that omission of the suicidality item has minimal impact on overall depression severity scoring, as this item is the least frequently endorsed in general population samples and contributes little to total score variance.^59–62^ Items assess symptom frequency over the prior two weeks and are rated on a 4-point scale from 0 (“Not at all”) to 3 (“Nearly every day”), with higher scores indicating greater depressive symptom severity.

During each 7-day burst period, participants completed a daily diary adaptation of the PHQ-8 assessing symptoms experienced since waking that day.^63^ Item selection and wording were informed by an established experience sampling repository (i.e., the ESM Item Repository) to ensure ecological validity and alignment with intensive longitudinal measurement practices.^64^ Items are presented in Supplementary Materials Table 1. The suicidality item was excluded due to safety monitoring constraints in unsupervised daily assessment contexts. To enhance conceptual specificity for network modeling, selected items were decomposed into theoretically distinct components. Specifically, the psychomotor agitation/retardation item was separated into two items distinguishing slowed movement/speech from restlessness, and the sleep item was divided to differentiate difficulty initiating or maintaining sleep (“trouble falling asleep or staying asleep”) from hypersomnia-related symptoms (“slept more than usual”). As a result of these modifications, the daily diary instrument included 10 depressive symptom items. Daily items were rated on a five-point scale ranging from 0 (“Not at all”) to 4 (“Very much”) and were administered at 8:00 pm, with automated hourly reminders at 9:00 pm and 10:00 pm before the survey closed at 11:00 pm. To support participant well-being, information about free university psychological services was provided upon completion of each daily diary.

#### Anxiety Symptoms (Secondary Outcome).

Anxiety symptoms were assessed given the high comorbidity and substantial symptom overlap between anxiety and depressive disorders,^65^ including evidence of interconnected symptom networks.^40,66^ Anxiety frequently co-occurs with depression during emerging adulthood,^67^ and symptom overlap may influence network dynamics and temporal associations with 24-hour movement behaviors. At baseline and monthly panel assessments, anxiety was measured using the 7-item Generalized Anxiety Disorder scale (GAD-7), a widely validated instrument with strong psychometric properties in college and emerging adult populations.^68^ Items assess symptom frequency over the prior two weeks using a 4-point scale from 0 (“Not at all”) to 3 (“Nearly every day”).

During each intensive burst period, anxiety symptoms were assessed daily using a 6-item adaptation of the GAD-7 referencing symptoms experienced since waking that day.^63^ Items are presented in Supplementary Materials Table 1. The item “Feeling so restless that it was hard to sit still” was excluded due to substantial conceptual overlap with the PHQ-8 psychomotor agitation item, thereby reducing redundancy across daily symptom measures and minimizing participant burden. Daily items were rated on a five-point scale from 0 (“Not at all”) to 4 (“Very much”).

#### 24-Hour Movement Behaviors (Primary Exposure).

Daily 24-hour movement behaviors were assessed using a wrist-worn Fitbit Charge 6 device provided at baseline and worn continuously (24 hours/day) throughout the 108-day study period. The device uses triaxial accelerometry and photoplethysmography-derived heart rate data to estimate time spent in sleep, SB, LPA, moderate PA (MPA), and vigorous PA (VPA) using proprietary algorithms. Daily aggregated time-based estimates for each intensity category were obtained via secure synchronization through the Fitbit mobile application and the Pathverse research platform. Daily time in sleep, SB, LPA, MPA and VPA will be used to derive 24-hour time-use compositions, with MPA and VPA combined into a single MVPA category for analysis.

Primary validation studies of Fitbit Charge and other wrist-worn Fitbit models demonstrate strong agreement with polysomnography for estimating total sleep time in young adults,^69,70^ and acceptable agreement with research-grade accelerometers for estimating time spent in intensity-based activity categories under free-living conditions.^71–80^ Although Fitbit devices rely on proprietary classification algorithms that likely reflect individualized (relative) rather than fixed absolute intensity thresholds,^81^ the present study emphasizes within-person day-to-day variation in 24-hour time-use distributions rather than absolute energy expenditure. Accordingly, minor classification error at specific intensity thresholds is unlikely to meaningfully bias inferences. Given the study’s focus on continuous day-to-day monitoring across a 108-day period, the Fitbit Charge 6 was selected as a practical, low-burden device capable of supporting continuous free-living assessment in emerging adults.

#### Daily Contextual Covariates (Burst Assessments).

During each burst period, participants completed additional daily items assessing contextual and affective factors experienced since waking that day. These items included: 1) Perceived stress; 2) Happiness; 3) Sleep quality (previous night); 4) Academic demands; and 5) Injury or illness status. Items were rated on a five-point scale from 0 (“Not at all”) to 4 (“Very much”), with the exception of sleep quality, which was scored from 0 (“Very poor”) to 4 (“Very good”). These variables were included as time-varying contextual covariates to account for daily influences on 24-hour movement behaviors and depressive symptoms. Items are presented in Supplementary Materials Table 1.

#### Daily Contextual Covariates (Weather).

Daily weather data, including high and low temperature, precipitation, humidity, and wind speed (average and max), were obtained retrospectively from the Kansas Mesonet station most proximal to campus (North Agronomy Farm) and linked to participant-level data by calendar date. These variables were assessed as time-varying environmental covariates.

#### Daily Contextual Covariates (Campus Recreation Center Usage).

Daily campus recreation center access data were obtained from university administrative records. Participants provided their student identification numbers at baseline, which were used to link recreation center entry records (via facility swipe access) to participant-level data by calendar date. Records reflected whether participants accessed campus recreational facilities on a given day. Facility usage was included as a time-varying covariate.

#### Monthly Psychosocial and Behavioral Measures.

At baseline and monthly panel assessments, participants completed validated measures assessing psychosocial, behavioral, and contextual factors relevant to mental health and PA. These included measures of loneliness,^82^ perceived stress,^83^ sleep quality,^84^ social support,^85^ college adjustment,^86^ stressful life events,^87^ body-related attitudes and behaviors,^88^ time spent in nature, and PA motivational constructs.^89–91^ A summary of panel measures, instruments, and assessment timing is provided in Table 3. A mid-study check-in survey at Week 8 assessed participant satisfaction, perceived barriers, technical issues, and suggestions for study improvement.

#### Baseline Demographic and Health Measures.

At baseline, participants reported demographic characteristics including age, sex, race/ethnicity, college of enrollment within the university, first-generation college status, parental education (proxy for socioeconomic status), residence hall, self-rated health, food security, and home ZIP code. Home ZIP codes were used to classify participants’ rural-urban residence status using established 2020 Rural-Urban Commuting Area (RUCA) codes.^92^

Height, weight, waist circumference, and hip circumference were measured during the laboratory visit. Height and weight were measured using a calibrated Health O Meter Professional 349KLX digital floor scale and stadiometer. Waist circumference was measured using a measuring tape placed midway between the lowest rib and the iliac crest while the participant was standing. Hip circumference was measured using a measuring tape placed at the level of the maximum circumference over the buttocks while the participant was standing with feet together and weight evenly distributed. Body mass index (BMI) was calculated as weight (kg)/height (m ). Waist-to-hip ratio (WHR) and waist-to-height ratio were computed to characterize central adiposity and abdominal fat distribution. Participants were classified as having abdominal obesity if their waist-toheight ratio was ≥ 0.50, consistent with established clinical cutpoints.^93^

Participants also reported current and past mental health diagnoses, psychiatric medication use, chronotype (i.e., individual differences in circadian preference for morning versus evening activity and alertness; Morningness–Eveningness Questionnaire),^94^ PA participation (Exercise Vital Sign),^95^ and resilience (Brief Resilience Scale).^96^ Participants provided personalized academic schedules (daily class hours), which were used to calculate instructional hours per day and identify the timing of key academic assessments (e.g., exams, major assignments). These data were incorporated into the daily time-series dataset to account for academic time demands and assessment timing.

#### Additional Health Measures (Follow-Up Assessment).

A follow-up health assessment visit which took place in the last three weeks of the study was added to the study protocol via amendment during the data collection period. At this visit, anthropometric measures (height, weight, waist circumference, and hip circumference) were repeated. Additional assessments introduced at follow-up included body composition, maximal handgrip strength, and resting blood pressure.

Body composition was assessed via bioelectrical impedance analysis using a Tanita DC-430U Multi-Frequency Total Body Composition Analyzer. Participants were instructed to empty their bladder prior to assessment. Maximal handgrip strength was measured using a Jamar Plus+ Digital Hand Dynamometer; participants completed three trials with their dominant hand, and the highest value was retained for analysis. Resting blood pressure was measured using an automated Omron Digital Blood Pressure Monitor (HEM-907XL) after participants rested quietly for two minutes. The average of two readings was recorded; a third measurement was obtained if the first two differed by ≥ 10 mmHg.

### Study Protocol

Eligible participants attended a baseline laboratory visit between August 25 and September 5, 2025. During this visit, trained research staff obtained anthropometric measurements, and participants completed baseline survey assessments. Participants were then provided with a Fitbit Charge 6 device, which was placed on their non-dominant wrist. Study staff assisted participants in downloading the Fitbit and Pathverse applications to their smartphones and guided them through secure linking and synchronization to enable data sharing with the research team. Participants were also randomized to one of six pre-specified intensive burst sequences (see Study Design) via study identification-level allocation. Specifically, study identification numbers were randomly assigned to the six sequences prior to enrollment, and participants were subsequently assigned to study identification numbers based on their selected scheduling timeslot.

Panel surveys were administered every four weeks following baseline via email and the Pathverse application. Participants were given five days to complete each panel survey. Each survey required approximately 20 minutes to complete and included three attention-check items to promote data quality. The intensive burst protocol commenced on September 2, 2025. Participants who enrolled after this date were randomized to their assigned burst sequence at baseline; for those assigned to Block S1 or S2 whose scheduled burst days occurred prior to enrollment, those days were not assessed and were coded as missing. During each 7-day burst period, daily surveys were delivered via the Pathverse application, with item order randomized at each administration. Surveys were released at 8:00 PM and remained accessible until 11:00 PM. Automated push notifications were sent at 8:00 PM, with reminder notifications at 9:00 PM and 10:00 PM if the survey remained incomplete.

Following the university’s fall break, participants were invited to return to the laboratory for a follow-up health assessment visit between December 1 and December 19, 2025. Repeat anthropometric measurements were obtained, and additional assessments included body composition via bioelectrical impedance analysis, maximal handgrip strength, and resting blood pressure (see Measures section for detailed procedures). Following study completion on December 19, 2025, participant engagement was reviewed, and compensation was calculated according to the pre-specified incentive plan. Digital gift cards were subsequently distributed via email.

### Compensation

Participants were eligible to receive up to $155 in compensation. Incentives were structured to promote sustained engagement across daily diary completion, device monitoring, and panel assessments. Participants received $1 per completed daily diary (maximum $35), with additional bonuses for achieving ≥ 50% ($15) and ≥ 80% ($15) diary completion. Incentives were also tied to Fitbit data synchronization thresholds based on valid wear days, defined as days with ≥ 1,000 recorded steps. Participants received $10 for syncing valid data on ≥ 50% of study days and another $10 for syncing ≥ 80% of study days. Participants received $10 for each completed monthly panel survey. An early engagement bonus ($10) was awarded to participants who synced valid Fitbit data on ≥ 80% of days (n = 22 days) during the first four weeks of the study. Participants received $10 for completing the second in-person assessment. Participants were permitted to keep the Fitbit device upon study completion.

### Data Analyses

#### Preparation.

Daily time-series datasets will integrate device-based 24-hour movement behaviors (sleep, SB, LPA, and MVPA), depressive symptoms, anxiety symptoms, contextual covariates, and academic load indicators. Prior to analysis, 24-hour movement behavior data will be inspected for zero values. Any observed zeros will be addressed using a log-ratio expectation–maximization (LREM) approach to permit valid compositional transformation.^97^ Daily wake-to-wake wear times will then be normalized to 1,440 minutes using the R package compositions,^98^ ensuring closure of the 24-hour composition. Movement behaviors will subsequently be transformed into isometric log-ratio (ilr) coordinates using sequential binary partitioning procedures.^99^ Four distinct sets of ilr coordinates will be constructed, each specifying a different behavior (sleep, SB, LPA, or MVPA) as the pivot coordinate (ilr ). This pivot approach enables examination of the relative importance of each behavior compared to the remaining components of the composition, while preserving the inherent co-dependence among sleep, SB, LPA, and MVPA.

#### Aim 1 (Within-Person Associations Between 24-Hour Movement Behaviors and Depressive/Anxiety Symptoms).

To examine dynamic longitudinal associations between specific depressive and anxiety symptoms and 24-hour movement behaviors, a series of multi-level vector autoregressive (mlVAR) models will be estimated.^100,101^ Each of the four time-use components that make up the 24-hour movement composition will be rotated into the ilr position to study its unique association (relative to time spent in other behaviors) in relation to each depressive and anxiety symptom. This framework allows for the simultaneous modeling of temporal (lagged), contemporaneous, and between-person associations while separating within-person dynamics from stable between-person differences.^102^ Time-lagged models will be used to examine whether day-to-day fluctuations in sleep, SB, LPA, and MVPA predict subsequent changes in specific depressive and anxiety symptoms, and vice versa. Centrality indices (strength, expected influence, betweenness, and closeness) will be computed to identify the most influential behaviors and symptoms within the network structure.

#### Aim 2 (Symptom Network Differences Across Behavioral Profiles).

Person-centered analytic approaches will be used to identify distinct 24-hour movement behavior composition profiles. Depending on distributional properties and model fit, approaches may include latent profile analysis, model-based clustering, or related mixture modeling techniques. Model selection will be guided by statistical fit indices (e.g., Bayesian Information Criterion), classification quality, theoretical interpretability, and class size considerations. Following profile identification, separate mlVAR models will be estimated within each behavioral subgroup to construct profile-specific symptom-behavior networks. Network comparison tests will be used to evaluate differences in global network strength, edge weights, and overall network structure between profiles.

#### Aim 3 (Periods of Heightened Risk for Symptom Onset and Progression).

To examine the temporal stability of the depression symptom network across the first college term, including 24-hour movement behaviors and anxiety symptoms as nodes, time-varying network analyses will be conducted using rolling 7-day calendar windows across the 108-day study period.^103^ Although participants contributed data during assigned burst weeks only, pooled daily observations across individuals provide coverage spanning the full academic term. Within each window, mlVAR models will be estimated to derive temporal (lagged) and contemporaneous networks while accommodating the unbalanced repeated-measures structure of the burst design. Window-specific network metrics, including global connectivity (e.g., average edge weight), network density, and node centrality indices, will be computed to evaluate changes in overall interconnectedness and node influence over time. Periods characterized by increased network connectivity or heightened centrality of specific symptoms or behaviors will be interpreted as potential windows of increased vulnerability – or, conversely, relative stability – during the transition to college. Calendar-based academic or institutional events (e.g., midterm periods, finals week, university-designated mental health initiatives such as Wildcat Pause Day) will be descriptively overlaid onto temporal network estimates to contextualize observed patterns.

## Discussion

The CALM Study was designed to advance our current understanding of how 24-hour movement behaviors influence depressive symptoms during the transition to college – a developmental window marked by peak risk for first-onset depression.^6^ By applying CoDA and mlVAR modeling to intensive time-series data collected across a 108-day academic semester, this study embeds rigorously modeled, modifiable behavioral exposures within symptom-level dynamic networks. This approach supports precision psychiatry by clarifying which 24-hour movement behaviors are most strongly linked to specific depressive (and anxiety) symptoms. The proposed study will also test whether distinct behavioral profiles demonstrate unique symptom network structures, and when these associations intensify during periods of heightened vulnerability.

For the field of behavioral medicine, a central contribution of the proposed work is its shift from modeling depression as a binary outcome or aggregate symptom score toward conceptualizing symptoms as components of a dynamic system. Although network approaches to mental illness have been well established in clinical psychology for over a decade,^104^ their integration into behavioral medicine has been comparatively limited, prompting calls to better integrate behaviors into network models of psychopathology.^44^ Traditional approaches can obscure heterogeneity in symptom presentation,^38,39^ and overlook the possibility that specific symptoms (e.g., fatigue, anhedonia, concentration difficulties) may be differentially linked to modifiable daily movement behaviors. For instance, insufficient sleep may be more proximally associated with fatigue or concentration problems,^105^ whereas prolonged SB may relate more strongly to anhedonia or low mood.^106^ Embedding 24-hour movement behaviors within symptom-level networks, therefore, enables examination of whether particular behaviors exert disproportionate influence on specific symptoms, and whether these behaviors function as influential perturbations within the broader depression network. Such distinctions are clinically meaningful, as they clarify early pathways of symptom activation and identify which modifiable behaviors most strongly destabilize or stabilize symptom networks over time – information that is central to advancing precision psychiatry and informing upstream intervention strategies.^107^

A further contribution of this study is its explicit modeling of 24-hour movement behaviors as a constrained composition. Because daily time is finite, any increase in sleep, SB, or PA necessarily displace time from other domains (e.g., increasing MVPA by 15 minutes reduces time spent in sleep, SB and/or LPA). Analytic approaches that treat these behaviors as independent exposures risk producing biased or misleading estimates.^29^ Although some studies in clinical psychology have begun incorporating behavioral variables such as sleep duration and exercise into symptom network models,^108–112^ these efforts have typically examined single behaviors in isolation, often relying on self-reported measures and without accounting for the constrained 24-hour structure of daily time use. Modeling 24-hour movement behaviors through a compositional lens, therefore, provides a more ecologically valid and statistically appropriate representation of how daily time-use distributions relate to symptom dynamics. By applying CoDA to wearable-derived time series behavioral data across an academic semester and integrating this framework with a network approach, the present study enhances the precision of estimated associations and allows examination of how within-person shifts in 24-hour movement behavior compositions temporally relate to fluctuations in symptom activation and network connectivity.

Beyond identifying behavior-symptom link specificity, an additional aim of this work is to determine whether shifts in behavioral time-use distributions precede periods of symptom escalation during the transition to college – a developmental window in which subthreshold symptoms may consolidate into clinically significant depressive episodes.^1,2,5^ Network theory suggests that systems may become more densely connected prior to transitions into more severe states,^113,114^ raising the possibility that time-varying symptom-behavior networks could signal emerging vulnerability. This perspective aligns with precision psychiatry initiatives such as WARN-D,^115^ which seek to detect early warning signals of depression using intensive longitudinal designs that integrate self-reports and passive sensing via wearable devices. At the same time, distinct 24-hour movement behavior composition profiles may demonstrate unique symptom-behavior network structures, suggesting that vulnerability processes differ across behavioral phenotypes. By modeling time-varying symptom-behavior networks across an academic semester, the CALM Study aims to evaluate whether changes in behavioral influence or overall network connectivity coincides with high-risk academic periods, and clarify both when and for whom behavioral perturbations are most strongly associated with symptom worsening. Although temporal associations do not establish causality, estimating lagged relationships at the daily level can generate hypotheses regarding modifiable behavioral processes that may destabilize or stabilize symptom networks during this critical developmental transition.

As with all longitudinal research in emerging adult populations, several limitations warrant consideration. First, the sample was drawn from a single university and was predominantly White, with relatively few first-generation status college students, which may limit generalizability to more diverse populations of emerging adults transitioning to college. Second, although wearable devices enable continuous free-living monitoring of 24-hour movement behaviors, reliance on proprietary heart rate-informed classification algorithms may introduce measurement imprecision relative to open-source processing of research-grade accelerometry. However, because the present analyses emphasize within-person associations, systematic classification differences are unlikely to meaningfully bias the primary inferences. Third, while mlVAR modeling allows estimation of temporal associations and separation of within- and between-person effects, these analyses do not establish causality; observed lagged relationships should therefore be interpreted as hypothesis-generating. Additionally, although the burst design enhances feasibility and reduces participant burden, intensive symptom assessment occurred during discrete weeks rather than continuously across the semester. As a result, short-lived fluctuations outside burst windows may not have been captured, and temporal inferences rely on pooled observations across individuals. Fourth, daily assessment of suicidality was excluded from the intensive burst protocol due to ethical considerations and the absence of real-time clinical monitoring during unsupervised reporting. Although necessary, this limited examination of the full range of depressive symptoms represented in the PHQ-9 and Diagnostic and Statistical Manual of Mental Disorders, Fifth Edition. Finally, sustained engagement with repeated panel surveys, intensive daily diary bursts, and continuous wearable monitoring across an academic semester may be challenging,^116^ particularly during periods of academic stress or schedule disruption. To mitigate potential attrition and response fatigue, the study incorporated a structured incentive framework, time-limited burst assessments designed to reduce burden, automated reminders within the mobile platform, and routine participant check-ins and personalized prompts delivered by the research team via email.

In summary, the CALM Study advances behavioral psychiatry by integrating compositional modeling of 24-hour movement behaviors with dynamic symptom network analyses during a critical developmental transition. By embedding rigorously modeled, wearable-derived behavioral exposures within time-varying symptom networks, this work moves beyond binary depression indicators and symptom severity sum scores toward identifying when and for whom specific behavioral perturbations are most strongly associated with symptom activation. Importantly, the hybrid panel-burst design not only enables high-resolution examination of short-term symptom-behavior dynamics but also provides a broader longitudinal framework for future exploratory prediction modeling of individual risk trajectories. Together, these insights lay the foundation for developing just-in-time adaptive interventions that leverage passive sensing to deliver personalized, behaviorally targeted support during periods of heightened vulnerability. As wearable technology becomes increasingly ubiquitous among emerging adults, integrating precision behavioral modeling with scalable digital intervention strategies holds promise for improving early detection and prevention of depression during the transition to college and beyond.

## Supplementary Material

Supplementary Files

This is a list of supplementary files associated with this preprint. Click to download.

- SupplementaryMaterialsTable1.DailyDiaryItems.docx

## Figures and Tables

**Table 1.** Design Constraints and Allocation Properties of the Intensive Burst

Design Constraint	Description
Exact global balance	Across all sequence assignments, Early (E), Mid (M), and Late (L) burst placements occurred equally often.
Exact per-period balance	Within each calendar period, two pre-specified burst sequence groups were assigned to each burst position (E, M, L), ensuring even distribution of assessment load across the five assessment windows.
Participant-level balance	Each sequence maintained a 2-2-1 distribution of burst timing (two assignments to two positions and one to the third), preserving comparable exposure patterns across participants.
Boundary control	Sequences were constructed to eliminate late-to-early transitions across adjacent periods, thereby preventing consecutive burst weeks at period boundaries. This constraint reduced the likelihood of back-to-back assessment weeks, helping to minimize participant burden and support sustained compliance across the semester.
Primacy and recency balance	Two sequences began and two ended with each burst position, reducing systematic timing bias.

**Table 2.** Cohort baseline characteristics.

Participant Characteristics	Mean (SD) or *n* (%)
Age (years)	18.2 (0.4)
Sex (Female)	79 (54.9%)
Race/Ethnicity	
Asian	2 (1.4%)
Black	3 (2.1%)
Hispanic	7 (4.9%)
White	113 (78.5%)
Multiracial	19 (13.2%)
First-Generation Status (Yes)	24 (16.7%)
Parental Education	
Less than high school	1 (0.7%)
High school	11 (7.6%)
Some college/Some University	13 (9.0%)
College/University	62 (43.1%)
Graduate/Professional degree	57 (39.6%)
College of Enrollment	
College of Health and Human Sciences	43 (29.9%)
College of Arts and Sciences	32 (22.2%)
College of Engineering	40 (27.8%)
College of Agriculture	14 (9.7%)
College of Architecture, Planning & Design	6 (4.2%)
College of Business Administration	5 (3.5%)
College of Education	4 (2.8%)
Self-Rated Health	
Fair	16 (11.1%)
Good	54 (37.5%)
Very Good	58 (40.3%)
Excellent	16 (11.1%)
Mental Health Condition Diagnosis	
None	116 (80.6%)
Current Diagnosis	23 (16.0%)
Single Diagnosis	8 (5.5%)
Two or More Diagnoses	15 (10.4%)
Anxiety Disorder	17 (11.8%)
Depression	14 (9.7%)
Attention-Deficit/Hyperactivity Disorder	10 (6.9%)
Autism Spectrum Disorder	3 (2.1%)
Obsessive-Compulsive Disorder	2 (1.4%)
Posttraumatic Stress Disorder	3 (2.1%)
Eating Disorder	2 (1.4%)
Bipolar Disorder	1 (0.7%)
Premenstrual Dysphoric Disorder	1 (0.7%)
Previous Diagnosis	3 (2.1%)
Depression	1 (0.7%)
Depression & Posttraumatic Stress Disorder	1 (0.7%)
Depression & Attention-Deficit/Hyperactivity Disorder	1 (0.7%)
Prefer Not to Say/Missing	2 (1.4%)
Psychiatric Medication Use	
Never	117 (81.3%)
Previous medication use	7 (4.9%)
Current medication use	18 (12.5%)
Prefer not to say/missing	2 (1.4%)
Depressive Symptom Severity (PHQ-8 total score)	3.20 (3.24)
PHQ-8 ≥10 (Clinically relevant depressive symptoms)	10 (6.9%)
Anxiety Symptom Severity (GAD-7 total score)	2.98 (2.30)
GAD-7 ≥10 (Clinically relevant anxiety symptoms)	1 (0.7%)

Note. Values are presented as mean (SD) for continuous variables and n (%) for categorical variables.

**Table 3.** Monthly psychosocial and behavioral measures details.

Measure	Primary Construct	Subdomains (if applicable)
*Assessed Every 4 weeks*		
UCLA Loneliness Scale (ULS-8)^82^	Loneliness	
College Student Acute Stress Scale^83^	Stress	2: Social and nonsocial stress
Pittsburgh Sleep Quality Index (PSQI)^84^	Sleep Quality	2 items: Sleep quality, sleep medication use
Multidimensional Scale of Perceived Social Support^85^	Social Support	3: Family, Friends, Significant Other
Eating Pathology Clinical Outcome Tracker (EPCOT)^88^	Eating Pathology	3: Body Dissatisfaction; Muscle Building; Binge Eating
Time Spent in Nature^117,118^	Time Spent in Nature	
Stage of Change for Physical Activity^119^	Stage of Change for Physical Activity	
Behavioral Intention^120^	Physical Activity Intentions	2: Decisional intention, Intention Strength
Physical Activity Regulation Scale^121^	Behavioral Regulation for Physical Activity	4: Proactive regulation, Reactive regulation, Social monitoring, Self-monitoring
Exercise Identity Scale^90,91^	Physical Activity Identity	Role identity
Self-Report Behavioral Automaticity Index^89^	Physical Activity Habit	
*Assessed Every 8 weeks*		
College Adjustment Questionnaire^86^	College Adjustment	3: Academic adjustment, Social adjustment, Psychological adjustment
College Student Stressful Event Checklist^87^	Stressful Events	

## Data Availability

Data is available upon reasonable request from the corresponding author.
